# A Spatial Transcriptome Reveals Changes in Tumor and Tumor Microenvironment in Oral Cancer with Acquired Resistance to Immunotherapy

**DOI:** 10.3390/biom13121685

**Published:** 2023-11-22

**Authors:** Yoh-ichiro Iwasa, Tomoyuki Nakajima, Kentaro Hori, Yoh Yokota, Ryosuke Kitoh, Takeshi Uehara, Yutaka Takumi

**Affiliations:** 1 Department of Otorhinolaryngology-Head and Neck Surgery, Shinshu University School of Medicine, Matsumoto 390-8621, Japan; k_hori@shinshu-u.ac.jp (K.H.); yywg@shinshu-u.ac.jp (Y.Y.); ryosuke@shinshu-u.ac.jp (R.K.); takumi@shinshu-u.ac.jp (Y.T.); 2 Department of Laboratory Medicine, Shinshu University School of Medicine, Matsumoto 390-8621, Japan; tnakajima@shinshu-u.ac.jp (T.N.); tuehara@shinshu-u.ac.jp (T.U.)

**Keywords:** head and neck cancer, immunotherapy, acquired resistance, immune checkpoint inhibitor, programmed death-ligand 1, digital spatial profiling

## Abstract

Although anti-programmed death-1 (PD-1) antibody therapy improves the prognosis in patients with head and neck squamous cell carcinoma (HNSCC), some patients exhibit disease progression even after showing a good response to the treatment initially because of acquired resistance. Here, we aimed to reveal the dynamic changes in the tumor and tumor microenvironment (TME) in a 77-year-old man diagnosed with oral squamous cell carcinoma who developed acquired resistance after the administration of nivolumab using spatial transcriptomics. The results showed that, before immunotherapy, the activated pathways in the tumor area were mainly related to the cancer immune system, including antigen processing cross-presentation, interferon–gamma signaling, and the innate immune system. After immunotherapy, the activated pathways were mainly related to epigenetic modification, including RMTs methylate histone arginine and HDAC deacetylates histones. Before immunotherapy, the activated pathways in the TME were mainly related to the metabolism of proteins, including SRP-dependent co-translational protein targeting the membrane. After immunotherapy, the activated pathways in the TME were related to sensory perception and signal transduction. Our study revealed that epigenetic-modification-related pathways were mainly activated after establishing acquired resistance, suggesting that epigenetic modification in the tumor may prevent cancer immune system activation via the anti-PD-1 antibody.

## 1. Introduction

Head and neck squamous cell carcinoma (HNSCC) is the sixth most common malignancy in the world. In the early stages, the long-term survival rate of patients with HNSCC is around 70–90%, whereas, in the advanced stages, there is a high risk of local recurrence and distant metastasis with a poor 5-year overall survival rate of <50% [1]. Before the immunotherapy era, platinum-based chemotherapy with anti-EGFR antibodies, such as cetuximab, was the standard therapy for recurrent and/or metastatic head and neck cancer (HNC) (R/M-HNC). Following the emergence of programmed death-1 (PD-1) antibodies, the treatment strategy dramatically changed and improved the prognosis of R/M-HNC in clinical settings. In the phase 3 Checkmate 141 study, nivolumab significantly improved the overall survival (OS) of patients compared to standard therapy [2]. In the phase 3 KEYNOTE 048 study, pembrolizumab alone significantly improved the OS compared to the cetuximab and chemotherapy combination in the combined positive score (CPS) of 20 or more populations and the CPS of 1 or more populations [3]. Based on the result of these phase 3 trials, nivolumab and pembrolizumab were approved in 2017 and 2019 in Japan and recognized as a first-line treatment for platinum-refractory HNC and platinum-sensitive HNC, respectively.

Although anti-PD-1 antibodies are promising therapeutic agents for R/M-HNC, the long-term survival of patients still remains 2 years, with an OS of approximately 20–30% [3,4,5]. Even in patients treated with pembrolizumab who initially show a complete response or partial response (PR), the median duration of response is under 24 months [6], which means that over 50% of patients treated show disease progression, even if they initially show a good response to the treatment, and this phenomenon is known as acquired resistance [7]. Similar treatment resistance has been observed in several cancers treated with different types of immune checkpoint inhibitors (ICIs). Although the mechanism of acquired resistance has been investigated, overcoming the resistance still remains a challenge.

**Figure 1:**
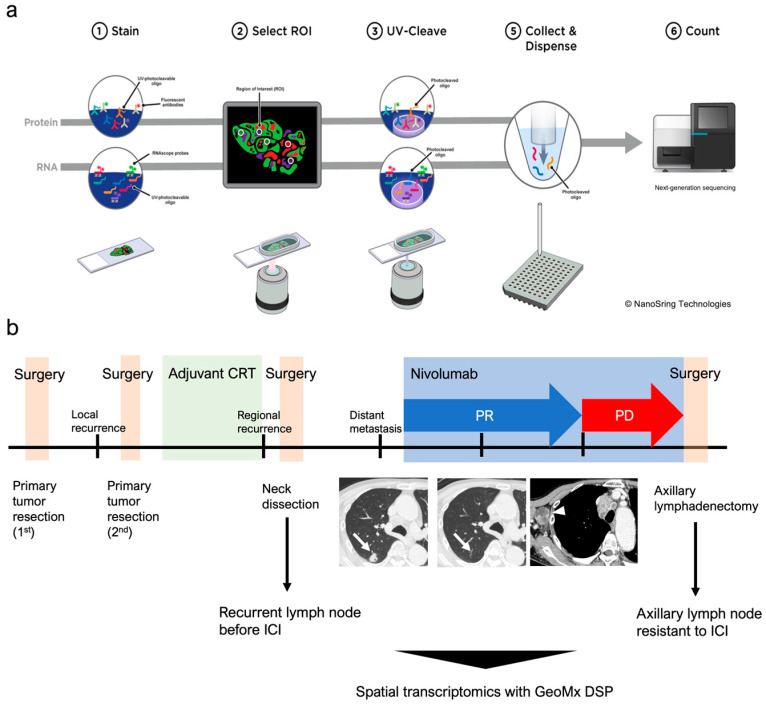
Spatial transcriptomics of oral squamous cell carcinoma treated with nivolumab. (**a**) Schematic representation of the spatial transcriptome with GeoMx DSP. This system is applicable to both RNA and protein. Tissue sections were stained using morphological markers for region of interest (ROI) selection. Photocleavage oligos from selected ROIs were processed for next-generation sequencing. (**b**) Schematic representation of the clinical course of the patient. Lung metastasis before the initiation of nivolumab almost disappeared (arrow). Lymph node metastasis was observed in the right-side axillary region (arrowhead) 13 months after initiation.

Recently, new technologies involving spatial transcriptomics, such as GeoMx DSP (NanoString Technologies, Seattle, WA, USA) and Visium spatial gene expression analysis (10× Genomics, Pleasanton, CA, USA), have allowed for the investigation of mRNA or protein expression in situ. GeoMx DSP, which was used in this study, enables the digital quantitative analysis of user-defined regions of interest (ROIs) in formalin-fixed and paraffin-embedded (FFPE) tissues (Figure 1a). The highly multiplexed spatial profiling technology has been applied in cancer research to identify predictive biomarkers and elucidate biological mechanisms in tumors [8,9]. Although GeoMx DSP has been used in a couple of studies on head and neck cancer [10,11], to our knowledge, no study has focused on the dynamic changes before and after acquired resistance. In this study, we used GeoMx DSP for the first time to investigate the dynamic changes in the tumor and tumor microenvironment (TME) of a patient with oral cancer who was treated with nivolumab and showed acquired resistance.

## 2. Materials and Methods

### 2.1. Patient Characteristics

This study involved a 77-year-old man diagnosed with oral squamous cell carcinoma (T2N0M0). After undergoing partial tongue resection twice, adjuvant chemoradiotherapy was administered. Owing to the immediate development of neck lymph node metastasis, right-side neck dissection was performed 2 weeks after CRT. Three months after the neck surgery, a CT-scan showed lung metastasis and lymph node metastasis in the mediastinal space and middle neck. Nivolumab was administered and a partial response was achieved during the first 10 months after treatment initiation (Figure 1b). Thirteen months after nivolumab initiation, lymph nodes in the right axillary region and mediastinal space were swollen, and pleural effusion was observed on the left side. Axillary lymphadenectomy was performed for definitive diagnosis, and the patient was diagnosed with lymph node metastasis.

### 2.2. Tissue Preparation and Digital Spatial Profiling

**Figure 2:**
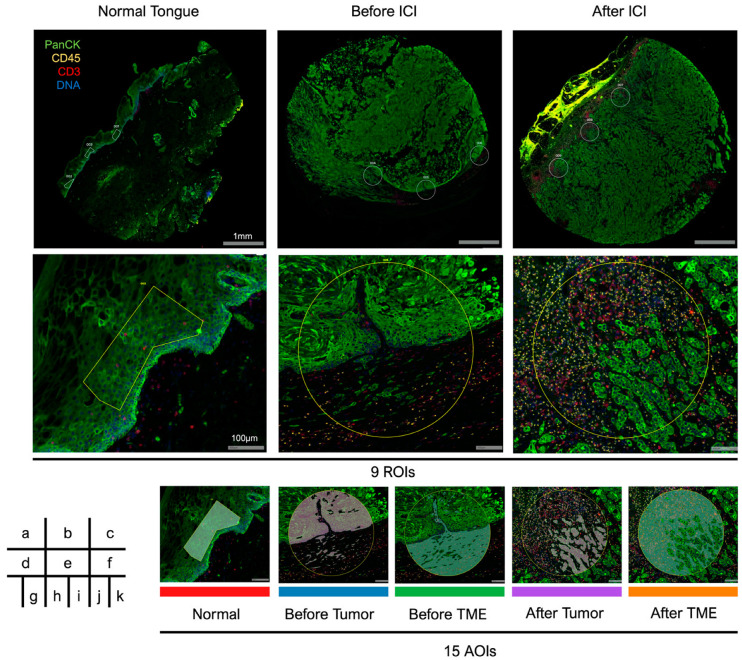
Fluorescent images of the analyzed tissue. (**a**–**c**) Low-magnification images of normal tongue tissue and lymph nodes before and after being ICI-trimmed using tissue microarrays. Regions of interest (ROIs) were selected at the tumor-infiltrating edge on each section. (**d**–**f**) High-magnification images of representative ROIs. (**g**–**k**) Each ROI was fractionated using PanCK+/−, and 15 areas of interest (AOIs) were independently analyzed.

Spatial gene expression was analyzed using the NanoString GeoMx platform as previously reported [12]. The FFPE tissues of metastasized neck lymph node were obtained before immunotherapy and the axillary lymph node was collected after the acquired resistance was cut to 5 μm thick sections and mounted on positively charged slides (VWR Superfrost Plus Micro Slide; 48311-703, Radnor, PA, USA). The slides were further processed by NanoString Technologies (Seattle, Washington, DC, USA). For morphological assessment, the sections were stained using fluorescently labeled reagents targeting pan-cytokeratin (AE1+AE3; Novus Biologicals, Centennial, CO, USA) to detect tumor compartments, CD45 (2B11+PD7/26; Novus Biologicals) for leukocytes, CD3 (UMAB54; Origene, Rockville, MD, USA) for T cells, and DNA (SYTO83; Thermo Fisher Scientific, Waltham, MA, USA). Whole transcriptome hybridization was performed using DNA oligonucleotides comprising 35 to 50 nt target-complementary sequences with the associated digital spatial profiling (DSP) barcodes. Fluorescent staining of the tissues distinguished the tumor infiltrating margin and associated TME (Figure 2), and ROIs for transcriptomic profiling were selected. Each ROI was segmented for PanCK+/− and illuminated using ultraviolet light, and the transcript-associated barcodes were collected for next-generation sequencing. Libraries were prepared according to the NanoString GeoMx-NGS Readout Library Prep manual. During library prep, each ROI collected from the tissue section was uniquely indexed using Illumina’s i5 × i7 dual-indexing system. In total, 4 μL of the aspirate containing the photocleaved oligos, 4 μL of primer from each well of the Seq Code Primer Plate, and 2 μL of 5X PCR MasterMix were mixed in a PCR plate and incubated in a thermocycler with the following program: 37 °C for 30 min, 50 °C for 10 min, 95 °C for 3 min, 18 cycles of 95 °C for 15 s, 65 °C for 60 s, 68 °C for 30 s, and a final extension of 68 °C for 5 min. PCR products were purified with two rounds of AMPure XP beads (Beckman Coulter, Sykesville, MD, USA). Sequencing was performed using NovaSeq 6000 (Illumina, San Diego, CA, USA). The resulting FASTQ files were then taken though the NanoString NGS Pipeline to obtain raw counts, which were Q3 normalized (third quartile; 75th percentile) prior to analysis. Data were analyzed using DSP Data Analysis Suite (NanoString Technologies).

## 3. Results

### 3.1. Fluorescent Staining and Selection of ROIs for Whole Transcriptome

Fluorescent images of the patient’s tissue stained with fluorescent antibodies are presented in Figure 2. Three ROIs were selected for each normal tongue tissue (ROIs 1–3), neck lymph node before ICI therapy (ROIs 4–6), and axial lymph node after ICI therapy (ROIs 7–9). Each ROI was fractionated using PanCK+/−, and each area was designated as an area of interest (AOI); PanCK+ areas were analyzed as tumors and PanCK− areas were analyzed as TME. These AOIs were analyzed independently using GeoMx DSP, and whole transcriptome analysis was performed using an Illumina sequencer. A total of 18,676 genes were assayed across 15 AOIs, 10,772 genes were expressed in 10% of AOIs, and 6222 genes were expressed in 50% of AOIs.

### 3.2. Differences in Gene Expression before Immunotherapy and after Acquiring Immunotherapy Resistance

**Figure 3:**
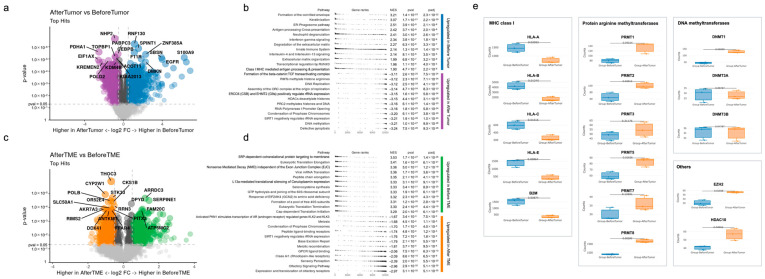
Spatial transcriptomics reveals dynamic changes in the tumor and tumor microenvironment during the development of acquired resistance to immunotherapy. (**a**,**b**) Volcano plots of significantly differentially expressed genes and the top 12 upregulated pathways in the Reactome pathway list in the tumor before and after immunotherapy. Violet: After Tumor; Blue: Before Tumor. (**c**,**d**) Volcano plots of significantly differentially expressed genes and upregulated pathways in the TME before and after immunotherapy. Orange: After TME; Green: Before TME. (**e**) Box plots showing expression levels of noteworthy genes in the tumor before and after immunotherapy.

We investigated gene expression differences before immunotherapy and after acquiring immunotherapy resistance. Volcano plots of significantly differentially expressed genes and upregulated pathways in the tumor and TME before and after immunotherapy are shown in Figure 3a–d. Pathway enrichment analysis was performed using Reactome database (version 78, September 2021) in GeoMx software. Before immunotherapy (Before Tumor), the upregulated pathways in the tumor area were mainly related to the immune system against cancer (Figure 3b), namely, antigen processing cross-presentation, interferon–gamma signaling, and class-1 MHC-mediated antigen processing and presentation. After immunotherapy (After Tumor), the activated pathways in the tumor were mainly related to epigenetic modification such as RMTs methylate histone arginine, HDAC deacetylate histones, PRC2 methylates histones and DNA, SIRT1 negatively regulating rRNA expression, and DNA methylation.

Before immunotherapy (Before TME), the upregulated pathways in the TME were mainly related to protein metabolism (Figure 3c): SRP-dependent co-translational protein targeting the membrane, eukaryotic translation elongation, peptide chain elongation, the L13a-mediated translational silencing of ceruloplasmin expression, GTP hydrolysis and the joining of the 60S ribosomal subunit, the formation of a pool of free 40 s subunits, eukaryotic translation initiation, and cap-dependent translation initiation. After immunotherapy (After TME), the upregulated pathways in the TME were related to sensory perception (olfactory receptor, olfactory signaling pathway, and sensory perception) and signal transduction (class A/1 (rhodopsin-like receptor), peptide ligand-binding receptors, GPCR ligand-binding receptors, and activated PKN1 stimulates the transcription of the AR (androgen receptor)-regulated genes *KLK2* and *KLK3*).

## 4. Discussion

ICI therapy has dramatically changed the treatment strategies for various cancer types since the approval of the CTLA-4 inhibitor for metastatic melanoma in 2011 [13]. Anti-PD-1 antibodies, namely nivolumab and pembrolizumab, have been approved for RM-HNC. Although anti-PD-1 antibodies have improved the prognosis in patients with R/M-HNC, they could be curative in only a small percentage of patients, indicating that the majority of the treated patients do not respond to the treatment. Furthermore, most of the patients who initially respond to the treatment later develop treatment resistance and show cancer progression. Resistance to ICIs is classified into two major types: primary resistance and acquired resistance [7]. Primary resistance refers to the clinical situation where a patient does not respond to ICIs. Acquired resistance refers to initial response to ICIs, followed by cancer relapse or progression after a while. The actual rate of acquired resistance has not been reported, but it is estimated to be 35–54% in patients with HNC [7]. Possible explanations for acquired resistance have been previously reported, including disruption or downregulation of antigen presentation, loss of interferon-γ sensitivity, neoantigen depletion, tumor-mediated immune suppression or exclusion, and additional inhibitory checkpoint use [7]. However, overcoming acquired resistance remains a challenge. Notably, in previous studies using bulk tumor tissue, transcriptome data have been obtained from whole tissues; therefore, the tumor and TME could not be distinguished. However, in this study, we performed a spatial transcriptomic analysis considering the difference between the tumor and TME. We noticed completely different mRNA expression profiles between these areas. This is the first study to evaluate the changes in mRNA expression in HNSCC before immunotherapy and after acquiring resistance to immunotherapy using spatial transcriptomics. We believe that our data reflect the actual changes in the tumor and TME during immunotherapy.

In this study, pathways related to the immune system against cancer, such as antigen processing cross-presentation, interferon–gamma signaling, and class-1 MHC-mediated antigen processing and presentation, were mainly activated in Before Tumor, and several epigenetic modification-related pathways were mainly activated in After Tumor. Therefore, epigenetic modification in tumors after ICI administration may hamper the efficacy of immunotherapy by obstructing immune pathways against cancer. Notably, the expression of genes encoding MHC class I molecules, such as human leukocyte antigen (HLA)-A, HLA-B, HLA-C, and HLA-E, and beta-2 microglobulin, significantly decreased in After Tumor (Figure 3e). Loss of MHC class I antigen presentation has been previously reported as a mechanism of acquired resistance to immunotherapy in melanoma and lung cancer [14,15]. Interestingly, epigenetic modification-related pathways detected in this study have been previously reported to be related to the aberrant expression of MHC class I molecules.

Regarding the pathway of RMTs methylate histone arginine, protein arginine methyltransferases (PRMTs) methylate arginine residues within proteins, thereby regulating several biological processes, such as epigenetic regulation, DNA damage response, and immune surveillance [16]. In this study, PRMT1, PRMT2, PRMT5, and PRMT8 were significantly highly expressed in After Tumor (Figure 3e). Inhibition of PRMT5, which methylates the histones H4R3me2s and H3R8me2s to repress gene expression, has been reported as a promising antineoplastic approach [17]. PRMT5 regulates antigen presentation by controlling NLRC5 expression, which regulates the expression of MHC class I genes, and the inhibition of PRMT5 expression leads to the increased expression of surface MHC class I and tumor cell recognition [18]. The inhibition of PRMT5 expression may enhance antitumor immune response, and the combination of PRMT5 inhibition and anti-PD-1 therapy may render resistant cold tumors susceptible to anti-PD-1 therapy.

EZH2 is the methyltransferase subunit of PRC2, which catalyzes the methylation of the histone H3K27, and the H3K27me3 modification can suppress the accessibility to chromatin and silence the expression of downstream genes [19]. As shown in Figure 3e, EZH2 expression in After Tumor significantly increased compared to Before Tumor. Our results showing increased EZH2 expression and decreased expression of MHC class I-related genes are consistent with those of a previous study, that is, EZH2 expression is negatively correlated with MHC class I antigen presentation [19]. The inhibition of EZH2 expression could be a promising therapeutic option for overcoming acquired resistance to immunotherapy in terms of the restoration of antigen presentation by class I MHCs. EZH2 inhibitors are currently being evaluated in clinical trials, such as a phase 1/2 study, using the combination of tazemetostat and pembrolizumab, involving multiple types of cancers, including HNSCC [20].

In the present study, two pathways, HDAC deacetylates histones and SIRT1 negatively regulates rRNA expression, were significantly upregulated. HDAC is divided into four types, and SIRT1 is classified as a class 3 HDAC. Histone deacetylation results in chromatin compaction, leading to transcriptional repression [20]. As the altered function of HDAC is closely related to the acquisition of malignant phenotypes in carcinogenesis, and the expression of several HDACs is upregulated during malignancies, HDAC inhibitors have been proposed for use in anticancer therapy [21]. A combination of HDAC inhibitors and ICIs was investigated in a phase 2 clinical trial (NCT0253851) [22]. In this clinical trial, pembrolizumab and vorinostat, which is a pan-HDAC inhibitor, were administrated to patients with R/M HNSCC and salivary gland cancer; in the HNSCC cohort, 32% patients showed PR and 20% showed stable disease (SD). This result was better than the PR rate of 20% in a historical control involving the single-dose administration of pembrolizumab. In the present study, the HDAC10 level significantly increased in After Tumor, and it has been reported as an epigenetic target in several malignancies [23].

Regarding the pathway of DNA methylation, three DNA methyltransferases (DNMTs), including DNMT1, DNMT3A, and DNMT3B, were mainly noticed. The methylation of a CpG dinucleotide in DNA by DNMTs plays multiple roles, such as silencing transposable elements, defense against viral sequences, and the transcriptional repression of certain genes, and cancer cells are characterized by aberrant DNA methylation [24,25]. In this study, the expression of DNMT1 significantly increased in After Tumor, whereas the expression of DNMT3A and DNMT3B did not change (Figure 3e). MHC class I-related gene expression is regulated by DNMT1 in human and mouse neuroblastoma cell lines [26]. Therefore, the increased expression of DNMT1 might have played a role in preventing antigen presentation in our patient by downregulating MHC class I-related gene expression, resulting in acquired resistance to immunotherapy. The administration of decitabine, a DNMT inhibitor, has been reported to increase neoantigen presentation by MHC class I in glioblastoma cells. A phase 1b clinical trial involving a combination therapy of decitabine and durvalumab for patients with R/M HNSCC is currently ongoing (NCT03019003). Notably, the primary objective of this clinical trial is the changes in the expression of HLA class I and tumor antigens. The restoration of MHC class I-related gene expression using inhibitors against DNMT may have the potential to overcome acquired resistance to immunotherapy.

Non-mutational epigenetic reprograming has recently been considered a hallmark of cancer [27]. It is thought to play important roles in tumor initiation and malignant transformation. Recent studies have reported that epigenetic changes are related to not only carcinogenesis, but also treatment resistance, including resistance to immunotherapy [24,28]. Epigenetic modifications occur in response to environmental changes such as hypoxia and inflammation [29,30]. Epigenetic changes in a patient, which may arise from environmental factors within the tumor, may downregulate antigen presentation in the tumors, resulting in acquired resistance. Given that epigenetic changes observed in our patient were not specific and involved several epigenetic alterations (histone methylation, histone deacetylation, and DNA methylation), a combination of several epigenetic modifiers could be therapeutically promising in overcoming acquired resistance. For instance, a study using DLBCL cell lines showed a synergetic effect of a combination of EZH2 inhibitor and decitabine, which is a DNMT inhibitor, on the expression of class I MHCs [31]. Treatment strategies with a combination of epidrugs according to the epigenetic profile are expected to be explored in the future, facilitating precision medicine.

In this study, the tumor-adjacent TME was examined independent of tumor cells. Upregulated pathways in After TME were mainly related to G protein-coupled receptors (GPCRs) such as the olfactory receptor, olfactory signaling pathway, class A/1 (rhodopsin-like receptor), and GPCR ligand-binding receptors. GPCRs are the largest and most diverse group of cell surface signaling receptors [32]. Recently, targeting GPCR has been reported to prevent and reverse cancer therapy resistance [33]. Olfactory receptors, which are GPCRS, are expressed in not only the olfactory epithelium but also various nonolfactory tissues, even in various types of cancer cells, and their expression levels have been reported to be related to the prognosis of malignancies [34]. The relationship between GPCR or olfactory receptors and immunotherapy resistance remains unclear, and further research is warranted.

Similar to our study, recent studies have focused on the changes that occur during cancer therapy. Luoma et al. performed single-cell RNA sequencing and a T cell receptor repertoire analysis of tumor and blood samples, collected prior to the treatment and during treatment, of patients with oral squamous cell carcinoma; the patients were treated with nivolumab monotherapy or a combination therapy of nivolumab and ipilimumab [35]. Based on the findings of the kinetic analysis of tumor-infiltrating and circulating immune cells, they showed that CD8-T cells with a tissue-residency program were early responders to ICI and that neoadjuvant ICI enhanced local and systemic tumor immunity. In another study, McNamara et al. performed multiplex spatial proteomic analysis with GeoMx DSP for HER2-positive breast tumors sampled pre-treatment and after HER2-targeted therapy. They demonstrated that on-treatment CD45 measurement robustly predicted pathological complete response [36]. Interestingly, neither pre-treatment protein expression nor bulk pre-treatment and on-treatment gene expression data established predictive features, suggesting that bulk tissue analysis or pre-treatment sample analysis are imperfect, and dynamic spatial analysis would be promising in this situation. Given that our study focused on the dynamic changes in tumor tissues during the treatment, we believe that it provides important insights into the mechanisms of acquired resistance to immunotherapy.

This study has some limitations. First, the lymph node specimens compared in this study were collected from different regions (neck region and axillary region); therefore, the differences in the sampling region may have affected the transcriptional analysis results. Second, the results of this study were obtained from just one patient; therefore, the results should be carefully interpreted, and they cannot be extrapolated to other patients with a similar type of resistance to immunotherapy. To extrapolate these conclusions to other patients, additional analysis of large cohorts of patients with oral squamous cell carcinoma developing acquired resistance to immunotherapy is warranted. Despite these limitations, it is worth noting that we were able to perform spatial transcriptome analysis with GeoMx DSP for the first time to grasp the dynamic changes in the tumor and tumor microenvironment separately.

## 5. Conclusions

We suggest that several epigenetic alterations may prevent the innate immune system from targeting cancer, resulting in acquired resistance, at least in our patient. The inhibition of epigenetic changes would be key to overcoming acquired resistance to immunotherapy.

## Data Availability

The data that support the findings of this study are available from the corresponding author on reasonable request.
